# Hospital Preparedness and SARS

**DOI:** 10.3201/eid1005.030717

**Published:** 2004-05

**Authors:** Mona R. Loutfy, Tamara Wallington, Tim Rutledge, Barbara Mederski, Keith Rose, Sue Kwolek, Donna McRitchie, Azra Ali, Bryan Wolff, Diane White, Edward Glassman, Marianna Ofner, Don E. Low, Lisa Berger, Allison McGeer, Tom Wong, David Baron, Glenn Berall

**Affiliations:** * McGill University, Montreal, Quebec; † North York General Hospital, Toronto, Ontario; ‡ Toronto Public Health, Toronto, Ontario; § Health Canada, Ottawa, Ontario; ¶ Mount Sinai Hospital, Toronto, Ontario, Canada

**Keywords:** Severe acute respiratory syndrome, outbreak, hospital, management

## Abstract

On May 23, 2003, Toronto experienced the second phase of a severe acute respiratory syndrome (SARS) outbreak. Ninety cases were confirmed, and >620 potential cases were managed. More than 9,000 persons had contact with confirmed or potential case-patients; many required quarantine. The main hospital involved during the second outbreak was North York General Hospital. We review this hospital’s response to, and management of, this outbreak, including such factors as building preparation and engineering, personnel, departmental workload, policies and documentation, infection control, personal protective equipment, training and education, public health, management and administration, follow-up of SARS patients, and psychological and psychosocial management and research. We also make recommendations for other institutions to prepare for future outbreaks, regardless of their origin.

On March 5, 2003, the first patient with severe acute respiratory syndrome (SARS) died in Toronto, Ontario, Canada. This index patient was a 78-year-old woman who, upon returning to Toronto from Hong Kong, transmitted the new variant coronavirus to her family (*1*). On March 7, her son was admitted to hospital, and he subsequently died on March 13. His unrecognized disease led to nosocomial transmission of this disease in Toronto (*1*). As of August 28, 2003, a total of 375 cases of suspected and probable SARS had been identified in Toronto; most of these cases occurred within healthcare facilities (*1*–*3*). A minority of cases were related to household and community transmission, most acquired after hospital visits. The last community-acquired case of SARS-associated coronavirus (SARS-CoV) infection was identified on April 13, 2003 (*2*).

On the basis of the absence of new cases two incubation periods after the last case, barrier precautions were downgraded in Toronto hospitals on May 8, 2003. However, on May 23, the medical community realized that nosocomial transmission of SARS to patients and visitors had been occurring on a single ward in North York General Hospital (NYGH) throughout April and early May (*3*). Staff had been protected by personal protective equipment and therefore, because of the absence of staff cases and an epidemiologic link, the identification of the cases was delayed.

On May 23, a second phase of the outbreak (SARS II) was declared at NYGH, and the hospital was designated as a level-3 institution, which indicated that SARS had been transmitted through unprotected exposure (*3*). Consequently, a 10-day work quarantine for all staff was imposed. While this action prevented a major staffing shortage, it required all staff to wear N95 respirators at all times in the facility. When not at work, staff were at home, in home quarantine. During SARS II at NYGH, 55 patients were admitted with a diagnosis of SARS, and another 200 patients were assessed in the emergency department. We discuss the multidisciplinary and cross-departmental response used to establish SARS care at NYGH and offer recommendations that may help other hospitals prepare for an outbreak of SARS or any other infectious agent.

## Building Preparation and Engineering

### Wards

At the peak of SARS II, NYGH had 46 patients with investigated, suspected, or probable SARS in respiratory isolation in private, negative-pressure rooms (*4*). This was accomplished because two nearly constructed, empty, hospital wings were available. Within 72 hours of the declared outbreak, two units were converted into SARS wards, one with 22 rooms, the other with 27. Each private, negative-pressure room had no drapes and contained minimal equipment: one chair, a bedside table, a hamper for discarded linen, a garbage bin for contaminated equipment, and a hand sanitizer. Outside each room, a table held the personal protective equipment for staff entering the rooms. Outside each SARS ward were change-rooms for staff to change in and out of scrubs at the beginning and end of each shift.

### Intensive Care Unit

We learned that the intensive care unit (ICU)’s capacity is one of the factors that governs the number of SARS patients a hospital can manage. Since approximately 20% of patients with SARS require ICU care, the maximum number of patients with SARS that a hospital can manage can be calculated (*5*). At NYGH, the ICU’s capacity was 22 rooms, which allowed the care of approximately 80 SARS patients in the hospital at any time. The ICU had private, self-contained, glass-enclosed rooms. The adjacent ward was a clean unit containing the standard ICU equipment as well as tables with personal protective equipment. Outside the ICU, a change-room was stocked with fresh scrubs and linen-disposal bins.

### Emergency Department and SARS Assessment Clinic

**Figure 1:**
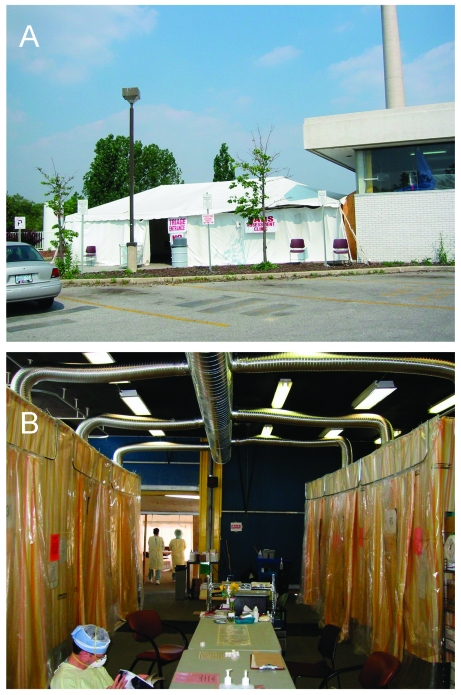
Tent assessment clinic built on ambulance loading dock for assessment of the general public for any symptom suggestive of severe acute respiratory syndrome. A, 40- x 20-foot tent constructed on the ambulance bay of the emergency department provided a spacious waiting area adjacent to the clinic area; B, inside the tent, eight cubicles were constructed with metal pipe frames and thick plastic walls, each ventilated with a custom-built ventilation system.

Similar principles were applied to the emergency department, which had eight private, negative-pressure rooms. This department was closed to the public because of the hospital’s level-3 status but stayed open for hospital employees and recently discharged patients. At the request of the provincial government, a SARS clinic was established to assess members of the public with symptoms of SARS. This clinic was constructed within 1 week in the 1,782–square foot ambulance bay. It contained eight negative-pressure isolation rooms built with pipe framing and plastic walls and ceilings. Areas for clerical work, registration, and changing personal protective equipment were also created. Other components included an area for case review, a lead-lined x-ray room, and an x-ray viewing room. A 40 x 20–foot tent was placed at the entrance of the clinic to provide ample space for a waiting area (Figure 1).

### Engineering and Maintenance

The above-mentioned wards were considered SARS units, and the same engineering principles were applied to each. Each patient’s room met the minimum requirement of six air changes per hour. Twice daily, the engineering department tested the negative-pressure status in all SARS units and patient rooms and presented the results to the hospital administration. In addition, an external company conducted daily assessments of the air circulation within the rooms. Highly trained engineering staff and clear blueprints and plans of the facility’s ventilation system were needed to implement all the required changes.

We recommend that hospitals take the following building preparation and engineering steps to prepare for an emerging infectious disease: 1) complete an assessment of their current facilities and capabilities; 2) ensure that current blueprints of the facility’s ventilation system are always accessible to facilitate expedient changes; 3) work with all relevant departments, including the proposed wards, ICU, and emergency department, to develop a strategy that allows for the rapid construction or conversion of the maximum number of private, negative-pressure rooms; and 4) identify in advance a timeline and areas of responsibility for constructing the maximum number of private rooms.

### Personnel

Our biggest challenge during the outbreak was insufficient personnel. Most personnel were required at the beginning of each phase and were then needed for approximately 3-1/2 weeks. Although more personnel were recruited, they did not start work for 1 to 2 weeks after the initial influx of patients. We required additional nurses, unit managers, infection control personnel, housekeeping, ward clerks, and supply stocking and inventory staff. Physicians recruited to manage the outbreak included primary-care doctors, infectious diseases consultants, hospital epidemiologists, public health physicians, emergency department physicians, and radiologists.

### Nursing Staff

In the SARS wards, we aimed for a high ratio of nurses to patients. At the beginning of the outbreak, the ratio was approximately 4–5 patients per nurse, a potentially dangerous ratio that could lead to transmission. During SARS II, the ratio was 1:1 if the patient was on oxygen requiring hourly monitoring and 2:1 for more stable patients. In the ICU, the ratio was two nurses per patient, which allowed for one nurse in the room and another outside. To avoid transmission, nurses were extensively trained in SARS patient care, the use of personal protective equipment, the potential risks for transmission, and preparedness for high levels of stress.

### Housekeeping

Dedicated and well-trained housekeepers were very important during the outbreak. Our housekeepers were trained in proper cleaning techniques and the use of personal protective equipment.

### Physicians

At NYGH, we used a most responsible physician (MRP) model for patient care, i.e., a primary care doctor (including emergency department physicians, general internists, family physicians, surgeons, and anesthesiologists who volunteered to care for SARS patients) directly cared for the patients. The patient-to-physician ratio for the MRP was 5–10 SARS patients per physician. One infectious disease consultant was assigned to each SARS ward, and one also covered the SARS ICU for a ratio of 20 to 30 SARS patients per infectious disease consultant. The MRP conducted all direct patient care, reviewed all cases, wrote the notes and, at mid-day, reviewed all cases with the infectious disease consultant, who was also responsible for alerting the MRP of new developments pertinent to SARS, for making changes in patient management, consulting with the emergency department for SARS assessments, and communicating with the onsite public health physician and outbreak management team. Training the emergency department physicians in SARS procedures was vital; our emergency department physicians became adept at evaluating potential SARS cases, which resulted in fewer patients being referred to infectious disease consultants.

We recommend that hospitals take the following personnel steps in advance of an emerging infectious disease event: 1) Calculate the maximum number of beds available for conversion to negative-pressure rooms on the wards, in the ICU, and the emergency department. The resulting figure will indicate the number of staff (including nurses, allied healthcare workers, and physicians) required from day 1. 2) Develop a system to identify those staff members who would be available to start working as part of the outbreak team within 24 hours. Such staff must be prepared for training and able to commit their services for a minimum of 3 to 4 weeks. 3) Generate a plan to meet the extra cost of hiring vital personnel (the greatest economic cost during such an outbreak). 4) Prepare for intensive training of both skilled staff and all other hospital employees in the use of personal protective equipment and infection-control procedures.

### Departmental Work Load

The SARS outbreak affected every hospital department. After NYGH was identified as a level-3 institution, only the two SARS units, the SARS ICU and the emergency department, continued to function. Most non-SARS patients were discharged, which left only 20 patients in this 400-bed hospital. However, every department’s continued contribution was needed. Occupational health played a major role in reviewing which healthcare workers could return to work. Environmental services and housekeeping were greatly affected by additional requirements throughout the hospital. Security ensured that unauthorized persons did not enter the hospital; a security staff member, with a nurse, escorted SARS patients on transports between departments, logging the date, time, and persons involved in the transfer. Diagnostic imaging staff and equipment were strained. Because of the isolation measures, SARS patients’ x-rays were taken with portable machines; two technicians were needed. The laboratory was overloaded due to the increased number of daily samples, which required a blood technician system to collect all samples at 7:00 a.m. Pharmacy staff handled increased orders and organized Health Canada’s approval requirements for ribavirin and interferon.

We recommend that hospitals conduct a review of each department’s existing capacity and capabilities for handling an outbreak. Strategies should then be developed to address any deficiencies.

### Policies, Procedures, and Documentation

During the outbreak, Toronto hospitals developed standardized systems for all implicated procedures, including code blues, patient transfers, and other infection-control procedures.

Of vital importance was the policy for patient oxygenation and early transfer to the ICU. SARS patients in need of oxygen can deteriorate rapidly, requiring intubations within 6 to 12 hours, a high-risk procedure that can lead to further nosocomial transmission (*6*). At NYGH, patients who had an oxygen saturation <92% and needed any amount of supplemental oxygen had their vital signs with oxygen saturation monitored every 2 hours instead of every 4 (*6*). If patients required more than 4 L/min of oxygen, the monitoring increased to every hour. Once patients required >6 L/min of oxygen, they were transferred to the ICU. Such early transfer allowed for elective, early intubation to be done in a controlled environment by minimal staff, which resulted in a reduced risk for transmission. Staff at intubations wore T4 Personal Protection Systems (Stryker Instruments, Kalamazoo, MI), although these items were not proven to have be beneficial (*6*).

Specific standardized forms were developed, including emergency department SARS consult sheets that included all the appropriate key questions regarding exposure, date of onset of symptoms, specific symptoms, laboratory investigations, and chest x-ray findings; admission order forms, which allowed for standard orders for the nurses and MRPs (see Appendix); and progress note forms, which documented symptoms, temperature, oxygen saturation and requirement, laboratory and x-ray results, and the daily plan. These documents both streamlined the process of daily review of 10 to 20 patients and standardized the level of care.

We recommend that hospitals do the following: 1) consider obtaining existing documentation and policies from other hospitals, such as NYGH; 2) develop an organized process of documentation that will facilitate an organized response to patient needs; and 3) assess different systems equivalent to the T4 Personal Protection Systems (Stryker Instruments) for particle removal efficiency and air-flow rate to choose the optimal system before an outbreak.

### Infection-Control Service

Before the SARS outbreak, NYGH had only two infection-control practitioners (ICPs). During the outbreak, additional ICPs were recruited, and hospital epidemiologists from other institutions arrived to create a system and infrastructure for infection control. We expanded an extant infection-control team to organize all the policies, systems, and structures for future infection control. Extra staff included a coordinator, four ICPs, a nurse clinician, a public health nurse, an administrator, a hospital epidemiologist (an infectious disease specialist with training in hospital epidemiology), and a clinical infectious disease physician. Members of this team made daily ward rounds to answer questions and conduct surveillance for fever and symptoms. In addition, they met several times a week to review policies, coordinate teaching, and address all other issues. To ensure consistent levels of infection-control practice, a system that reviewed the quality of practice was established: it was essential for the ICPs to maintain some degree of authority on these issues.

Because infection-control issues are vitally important, a hospital should do the following: 1) identify an appropriate number of ICPs for the hospital size (*7*); 2) include a qualified hospital epidemiologist on the infection-control team; 3) include a public health physician or designate on the team; 4) maintain constant vigilance during symptom surveillance; 5) sustain excellent standards of staff training and communication and apply continuous monitoring of infection-control practices; 6) ensure that all policies and documentation go through this team; and 7) provide the team with the necessary authority to work effectively throughout the hospital.

### Personal Protective Equipment and Fit-Testing of Respirators

The constant availability and use of personal protective equipment (much of which was disposable) was essential during the outbreak, including the following: N95 respirators, goggles, face shields, hair nets, gowns, and scrub suits. Specific policies and procedures were developed for putting on and removing personal protective equipment. For example, before entering a SARS patient’s room, a staff member wore an N95 respirator, goggles, face shield, hair net, a gown over scrubs, and two pairs of gloves. The order in which personal protective equipment was removed when a staff member exited a patient’s room was exact. For example, inside the room by the door, the first pair of gloves was removed, followed by the hair net, the face shield, and the second pair of gloves; next, hands were washed with quick-drying antiseptic solution, and the gown was carefully removed; then the hands were washed again before the staff member left the room. In the hallway, hands were washed, goggles removed and disposed of, hands washed again, respirators removed, hands washed, and finally, a new N95 respirator was donned. During SARS II, the provincial government issued a directive requiring that respirators be fit-tested at all hospitals. This recommendation proved to be difficult to implement at NYGH because we were in the midst of an outbreak.

We recommend that, in advance of an outbreak, hospitals should do the following: 1) prepare clear policies on the proper use of personal protective equipment during such an outbreak; 2) ensure that adequate supplies of essential items required for personal protective equipment will be available or are already stocked; 3) have staff fit-tested for respirators and document results or obtain forms of higher level respiratory protection that do not require fit-testing (e.g., loose-fitting powered air-purifying respirators).

### Education and Training

All staff had to be trained and educated on every aspect of SARS, including the proper use of personal protective equipment, risks to themselves and their families, and infection-control policies and procedures. At NYGH, training was conducted by a group of nurse clinicians assigned to each unit. Daily full-day, mandatory training sessions for all staff working on the SARS wards were created and included topics such as the proper way to don personal protective equipment, the psychological impact of SARS, and general infection-control practices.

We recommend that hospitals do the following: 1) include all departments in training, preferably in advance of an outbreak; 2) develop a program for certification in “Readiness to Manage an Infectious Medical Disaster Outbreak”; 3) develop a “train-the-trainer” model together with continued quality assurance monitoring.

### Public Health Outbreak Management Team

One unique feature of this outbreak was the formation of a mobile public health outbreak management team. It included two physicians, a manager, and five investigators (either public health nurses or inspectors), who were stationed beside the hospital coordinators and infectious disease specialists and remained onsite 24/7 for 4 weeks. This setup promoted outstanding communication and excellent relations between all parties, which allowed rapid exchanges of information that led to swift contact tracing and the quarantine of persons identified as having had unprotected exposure to a SARS patient. The public health nurses attended morning ward meetings to review management plans for patients admitted overnight, followed patient progress directly on the wards, and attended the regular infection-control team meetings.

We recommend that the healthcare system do the following during an outbreak: 1) facilitate effective communication between public health and hospital staff; and 2) establish a common information technology platform that allows for a streamlined, accessible flow of data between jurisdictions.

### Management and Administration

At NYGH, a 24-hour command center administered all the details connected to managing the outbreak and answered all questions. Department heads met daily at 9:00 a.m., which allowed them to impart important information to their staff. At 11:00 a.m., the SARS management committee held a meeting at which all decisions for the hospital were subsequently implemented. The key frontline players—including unit managers from each ward, infection control, infectious disease, and the chief of medicine—met daily to exchange information and properly manage the outbreak. Forums were regularly held by the hospital president to answer questions from the staff. All media contacts went through the single public relations department, to transmit a single message during this controversial time. We recommend that hospital administrations be prepared to play a pivotal role during such an outbreak.

### SARS Follow-up Clinic

**figure 2:**
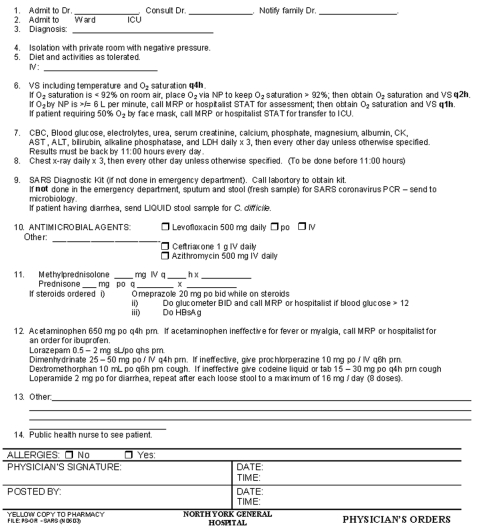
Standardized follow-up checklist of patients with severe acute respiratory syndrome. BP, blood pressure; CBC, complete blood count; LDH, lactate dehydrogenase; AST, aspartate transaminases; PCR, polymerase chain reaction; RR, respiratory rate; P, pulse; ALP, alkaline phosphatase; CK, creatine kinase; ESR, erythrocyte sedimentation rate; Ca, calcium; PO4, phosphate; Mg, magnesium; F/u, follow-up; NP, nasopharangeal; PA, posterior-anterior.

Patients recovering from SARS were discharged to remain in quarantine at home for an additional 7–10 days. Follow-up appointments were made for days 11 and 30 postdischarge. A single physician coordinated and ran the SARS follow-up clinic out of the emergency department. Again, a list of standard, step-by-step procedures was made for the assessment, and a checklist was designed (Figure 2). The physician assessed symptoms and reviewed follow-up laboratory tests. Convalescent-phase serologic tests were conducted 3–4 weeks after the onset of symptoms, and if results of a polymerase chain reaction test were positive for SARS-CoV, the test was repeated. Follow-up chest x-rays and, occasionally, computed tomographic scans were performed. A psychologist and a social worker provided psychological assessment and support during the follow-up visits. For SARS follow-up clinics in other hospitals, we recommend that, using standardized and organized methods, the hospitals prepare plans for an extensive follow-up system.

### Psychological and Psychosocial Management

Psychological and psychosocial support for both patients and the entire hospital staff was necessary during the SARS outbreak. Staff were affected by the fear of contracting and transmitting this new disease; SARS patients experienced stress because of their isolation, fear for their lives, guilt, anger, anxiety, and depression.

At NYGH, we put together a SARS psychological team (including social workers, psychiatric crisis nurses, psychiatrists, and infectious disease specialists) that developed a plan to manage the psychological impact on patients and staff. Patients were seen at least twice weekly. A social worker phoned each patient on days 2 and 6 postdischarge to follow up. A psychiatric crisis-line phone number was given to every patient in case he or she needed urgent attention. An outpatient system with psychiatrists was put in place to handle posttraumatic stress syndrome. These services were also established for all hospital staff. A quiet staff room was available for relaxation or discussion with a team member. After the outbreak, debriefing sessions were held with trained psychologists and counselors. We recommend that hospitals’ psychiatry departments, in conjunction with their hospital administration, develop a response plan for a crisis outbreak.

## Research

Research is imperative during such an outbreak, particularly for a new disease. The physicians and staff who were managing the outbreak had minimal time to do research, but they had many urgent questions. At NYGH, infectious disease and internal medicine physicians from Health Canada, Toronto Public Health, and other organizations came in to help with the research. The ethics board was prompt in attending to required approvals, often a lengthy process. Funding for such emergency research—another important factor—was provided.

To facilitate emergency research, we recommend that hospitals do the following: 1) identify potential members for collaborative research before an outbreak; 2) establish an expedient process for ethics approval; and 3) be prepared to alert funding agencies for the need for additional funding and support.

## Conclusion

A multidisciplinary approach to manage the second phase of the SARS outbreak in Toronto was undertaken at NYGH. This successful approach was only possible with the hard work and collaboration of many people as well as open and active communication maintained among all departments, employees, and patients. Many lessons, taken from this experience, can be applied by hospitals preparing themselves for such an outbreak. Finally, the policies, procedures, and documents developed at our institution and others are freely available to other centers to review and adapt as appropriate.

## Supplementary Material

Appendix Sars Outpatient Follow-Up Protocol
