# Supplementary material for: Hospital Preparedness and SARS
Source: Emerg Infect Dis. 2004 May;10(5):771–6. doi: 10.3201/eid1005.030717 (PMC3323236; doi:10.3201/eid1005.030717)
Supplement: Appendix — Sars Outpatient Follow-Up Protocol [file 03-0717-app-s1.pdf]

Appendix

SARS OUTPATIENT FOLLOW-UP PROTOCOL

- Nursing assessment
- Temperature: **oral**
- BP, RR, P
- O_2 saturation

Laboratory work:

- CBC, urea, creatinine, electrolytes, glucose, LDH, AST, ALP, bilirubin, albumin, CK, C Reactive Protein, ESR, Ca, PO_4, Mg
- One red-top tube for SARS serology, *Mycoplasma*, *Legionella*, *Chlamydia*
- F/u SARS diagnostic kit:
    - ❑ NP swab in viral transport medium (PCR)
    - ❑ Nasal swab in viral transport medium (PCR)
    - ❑ Throat swab in viral transport medium (PCR)
    - ❑ Rectal swab in viral transport medium OR stool specimen in sterile container (PCR)
    - ❑ Urine specimen in sterile container (PCR)

- CXR: portable PA
- MD assessment
- Social work
- Psychology

PRN:

- Crisis team
- Psychiatry
- Public health

Appendix. Standardized admission orders for patients with severe acute respiratory syndrome. A hospitalist is a general internist who works in the hospital covering the war and intensive care unit; CBC, complete blood count; AST, aspartate transaminases; ALT, alanine transaminases; LDH, lactate dehydrogenase; HBsAg, hepatitis B surface antigen; prn, pro re nata, i.e., when necessary; q4h, every 4 hours.
